# Medial patellofemoral ligament (MPFL) reconstruction in combination with a modified grammont technique leads to favorable mid-term results in adolescents with recurrent patellofemoral dislocations

**DOI:** 10.1007/s00167-017-4425-x

**Published:** 2017-02-16

**Authors:** Helmut Wegmann, Christoph Würnschimmel, Tanja Kraus, Georg Singer, Robert Eberl, Holger Till, Matthias Sperl

**Affiliations:** 1 Department of Paediatric and Adolescent Surgery, Medical University of Graz, Auenbruggerplatz 34, 8036 Graz, Austria; 2 Department of Paediatric Orthopedics, Medical University of Graz, Graz, Austria

**Keywords:** Patellofemoral instability, Trochlear dysplasia, Tibial tuberosity trochlear groove, Grammont technique, Medial patellofemoral ligament

## Abstract

**Purpose:**

The aim of the present study is to present the outcome of a cohort of adolescent patients with trochlear dysplasia and elevated tibial tuberosity trochlear groove (TTTG) distance suffering from recurrent patellar dislocation. Treatment consisted of medial patellofemoral ligament (MPFL) reconstruction and a modified Grammont procedure.

**Methods:**

MRI examinations were obtained pre- and postoperatively. Trochlear dysplasia was classified according to Déjour, and TTTG was measured on MRI. The Tegner Activity Scale and the Kujala Knee Score were assessed preoperatively and at follow-up. The Kujala Knee score and the IKDC 2000 knee score were documented at follow-up (median 50, range 20–61 months; SD 16.6).

**Results:**

Seven knees of six patients (median age 16.5 years, range 14–17 years) with trochlear dysplasia and elevated TTTG distance (median 17 mm, range 16.1–21.9 mm; SD 2.8) were treated. Trochlear dysplasia was classified as Déjour type A in 1, type B in 5, and type C in 1 knee. The Kujala Knee Score significantly increased from values of 55 (range 17–88; SD 25.9) to 94 (range 73–100; SD 9.1) at follow-up (*p* = 0.028). TAS improved from preoperative 2 (range 0–7; SD 2.5) to 5 (range 4–9; SD 1.8) at follow-up (*p* = 0.034). Median IKDC 2000 Knee Score at follow-up was 89 (range 61–100, SD 13.4). No re-dislocations were encountered.

**Conclusion:**

In selected adolescents with recurrent patellofemoral instability, MPFL reconstruction in combination with a modified Grammont technique yields excellent functional outcome and could, therefore, help to avoid major procedures, such as osteotomies.

**Level of evidence:**

Therapeutic, Level IV.

## Introduction

The stability of the patellofemoral joint is guaranteed by a complex interaction between the surrounding soft tissue and bony structures. Active stabilizers (the quadriceps muscle) can be differentiated from passive stabilizers, such as the retinacula and the medial patellofemoral ligament (MPFL), and static stabilizers, such as the trochlear geometry itself [30]. The importance of these stabilizers is underlined by the fact that the MPFL is ruptured in nearly all cases of patellofemoral dislocation [24] and that 96% of patients with a history of a patellar dislocation have evidence of trochlear dysplasia [11]. In addition, trochlear dysplasia seems to be a major risk factor for failure of operative stabilization of recurrent patellofemoral instability (PFI) in children and adolescents [22]. Another relevant anatomical factor responsible for recurrent instability is an increased distance between the tibial tuberosity and the trochlear groove (TTTG). The TTTG distance has been shown to be abnormal in more than half of the cases with PFI as compared to 3.5% in control knees [11]. Therefore, an increased TTTG distance in patients with PFI represents an indication for a medialization of the tibial tubercle [8, 11, 26].

A plethora of different surgical techniques has been described for addressing PFI [19]. In children, the surgical procedures historically involved a combination of lateral release, medial reefing, and the Roux–Goldthwait procedure [22]. However, these procedures do not adequately address the underlying pathology, and high failure rates have been reported [34]. Additional negative side effects of the Roux–Goldthwait procedure and tibial tuberosity transfer include increased patellofemoral pressure and patellar tendon shortening [1, 17]. Trochleoplasty can only be performed after closure of the growth plate of the distal femur [22], while reconstruction of the MPFL, as an anatomic procedure, has been advocated as the treatment of choice in skeletally immature patients [5, 9, 31]. Grammont and coworkers have described a technique for patella realignment consisting of a lateral release and medial fixation of the patella tendon to the tibial tubercle [13]. Kraus et al. have reported a modification of the Grammont technique, with lateral release of the patella tendon but without fixation of the patella tendon to the medial tibial tubercle. However, a reconstruction of the MPFL was not performed, and in patients with higher grade patella-femoral dysplasia, a significant rate of re-dislocations was found [15].

The combination of MPFL reconstruction and a modified Grammont technique could avoid major operative procedures, such as trochleoplasty or transfer of the tibial tuberosity in adolescent patients with trochlear dysplasia, elevated TTTG, and recurrent patellar dislocations. However, its efficacy has not been reported before. We hypothesized that performing this less extensive technique effectively addresses PFI and avoids re-dislocations.

## Materials and methods

Between 2009 and 2013, seven knees of six patients (*n* = 3 females, *n* = 3 males, one male bilaterally) were treated with a combination of MPFL reconstruction and a modified Grammont technique. All patients suffered from recurrent patella dislocations following unsuccessful conservative therapy with splinting followed by muscle strengthening.

Preoperatively, trochlear dysplasia was classified according to Déjour into types A–C. The Tegner Activity Scale and the Kujala Knee Score were assessed [16, 33]. Radiographs of the affected knees were obtained in a.p., lateral and merchant view, and patellar height was measured according to Caton-Deschamps [7]. In addition, tibial tuberosity trochlear groove (TTTG) distance and trochlear dysplasia according to Déjour [10] were measured on preoperative MRIs.

All procedures were performed by two senior surgeons. The first step consisted of a soft-tissue release of the patellar tendon applying the modified Grammont technique as described by Kraus et al. [15]. Briefly, the patellar tendon and the insertion of the patellar ligament at the tibial tuberosity were exposed using a lateral approach. The subcutaneous tissue along the tibial crest was bluntly mobilized. The patellar tendon was then sharply dissected and separated from the tibial tuberosity. After mobilization of the patellar tendon, it still remained attached to the distal periosteum. Subperiostal mobilization with a rasp was performed from the tibial tuberosity to the midshaft of the tibia. In knee flexion, the patellar tendon was then allowed to spontaneously slide medially to track within the femoral groove. In contrast to the original technique described by Grammont et al. no additional fixation of the patellar tendon to the tibia was performed [13]. Thereafter, MPFL reconstruction was performed as proposed by Schoettle et al. in 2009 using the gracilis tendon [29].

Postoperatively, all patients underwent a standard rehabilitation protocol consisting of isometric muscle activation 2 days after surgery and continuous passive motion with a range of 30°–60° of knee flexion starting on the third postoperative day.

All patients were invited for a follow-up examination. Clinical examination consisted of an apprehension test, an examination for tenderness, and range of motion of the affected knee. TAS, the Kujala Knee score and the IKDC 2000 knee score were documented. Radiographs of the affected knee were obtained in a.p., lateral and merchant view. A postoperative MRI was performed in all patients. In addition, the patients were asked whether or not they would again opt for an operative treatment.

The study was approved by the ethics committee of the Medical University of Graz (ID: 26-555ex13/14).

### Statistical analysis

Comparison of the preoperative and postoperative values was performed with the Mann–Whitney *U* test. Data are displayed as medians [range and standard deviation (SD)]. A *p* value of <0.05 was considered statistically significant.

## Results

Median age at surgery of the patients was 16.5 years (range 14–17 years, SD 1.3). All patients had functionally closed physes of the distal femur and showed signs of anatomical predisposition to patellofemoral instability. Trochlear dysplasia Déjour type A was diagnosed in one knee, Déjour type B in five knees, and Déjour type C in one knee. TTTG distances were elevated in all patients (median distance: 17 mm, range 16.1–21.9 mm, and SD 2.8). Patellar height according to the Caton-Deschamps index was normal in all patients (median 1.2, range 1.0–1.3, SD 0.1). Median sulcus angle was 144 (range 138–160; SD 8.9).

Follow-up examination was performed after a median of 50 months (range 20–61 months, SD 16.6 months). The Kujala Score and the Tegner Activity Score significantly improved comparing pre- and postoperative values (Table 1). Patellar height at follow-up did not differ from preoperative measurements. The median IKDC 2000 knee score at follow-up was 89 (range 61–100, SD 13.4). Postoperative MRI examinations showed a significant medial shift of the distal patellar tendon in the axial plane.

**Table 1:** Kujala Score and Tegner Activity Score preoperatively and at follow-up

	Preoperative [median (range, SD)]	Follow-up [median (range, SD)]	*p* value
Kujala Score	55 (17–88, 25.9)	94 (73–100, 9.1)	0.028
TAS	2 (0–7, 2.5)	5 (4–9, 1.8)	0.034

Median follow-up 50 months of seven knees in six patients undergoing a combination of MPFL reconstruction and a modified Grammont technique due to recurrent patellar dislocations

*SD* standard deviation, *TAS* Tegner Activity Score

In the follow-up period, no patellar re-dislocation occurred. One patient with Déjour type B trochlear dysplasia and 20 episodes of patellar dislocation prior to surgery showed a positive apprehension test at 30° flexion, but denied problems at daily activities. All patients stated that they would opt for an operative correction again.

## Discussion

The most important finding of the present study is that a combination of MPFL reconstruction and a modified Grammont technique leads to favorable mid-term outcome in selected adolescent patients with recurrent patellofemoral dislocations. At a median follow-up of 50 months, no re-dislocations occurred and the Kujala Knee Score and the Tegner Activity Score significantly improved comparing pre- and postoperative values.

Dislocations of the patella usually occur in children and adolescents [23]. For acute patellar dislocations, conservative management consisting of early joint motion and quadriceps strengthening following initial long-leg casting or bracing is the treatment of choice [32]. Nevertheless, in patients with PFI and predisposing anatomical geometry conservative treatment fails in almost half of the cases [4, 32]. Therefore, operative interventions are recommended in patients with recurrent patellar dislocations to prevent unfavorable long-term sequelae, such as chondral damage and subsequent osteoarthritis [18].

Conservative treatment failed in all adolescents in our series and the patients suffered from recurrent patellar dislocations justifying a surgical approach. Several different surgical procedures for the treatment of recurrent patellar dislocations have been described with variable results. Since the MPFL represents the main constraint against patellar dislocations [2, 25], MPFL reconstruction has been established as an effective treatment against repetitive patellar dislocations [12, 20]. However, in the presence of predisposing anatomical geometry, such as trochlear dysplasia or increased TTTG distance, MPFL reconstruction alone might be insufficient [22].

There are contradicting results for bony medialization of the tibial tuberosity as a stand-alone procedure in patients with increased TTTG distance. Therefore, some authors have combined bony tibial tuberosity transfer with MPFL reconstruction in patients with elevated TTTG and have reported a more favorable outcome [6, 29]. However, bony medialization of the tibial tuberosity should not be performed in patients with open physes. In addition, long-term follow-up has been shown to lead to premature arthritis of the medial knee compartment due to changes of the surface pressure values [14, 21]. In addition, there is no study relating the TTTG distance to the height of the patient, and therefore, no true cutoff for pathologic values of the TTTG distance exists. Consequently, it is difficult to decide how much correction of the TTTG should be performed. The “soft tissue tibial tuberosity osteotomy”, reported by Schneider and coworkers, represents an alternative technique yielding good functional outcome without growth disturbances after 4 year follow-up in patients with patellofemoral instability [28]. In 2012, Kraus and coworkers have reported good functional outcome of this technique in skeletally immature patients. The authors stated that restoration of the alignment of the distal patellar tendon by dynamic positioning represents a feasible method to treat recurrent patellar dislocation in skeletally immature patients. Nevertheless, patients with a higher grade of trochlear dysplasia (Déjour type B and C) showed a tendency to re-dislocate [15].

Preoperative evaluation revealed predisposing trochlear dysplasia and elevated TTTG distance in all our patients. Consequently, applying the two above-mentioned methods as stand-alone procedures might have been insufficient for these patients. Therefore, we have decided to apply a combination of the modified Grammont technique and MPFL reconstruction.

Treatment of PFI necessitates an individually tailored approach to correctly address the underlying pathologies. Our patients presented with insufficient MPFL, elevated TTTG distance as well as trochlear dysplasia. While we have tackled the first two pathologies with MPFL reconstruction and the modified Grammont technique, we are aware that performing an additional trochleoplasty would have been a treatment option for our patients with functionally closed physes. There are reports with promising mid-term results of primary trochleoplasty in patients with PFI and trochlear dysplasia [3]. Nevertheless, a recently published report by Rouanet and coworkers presents the outcome of 34 trochleoplasties at a mean follow-up of 15 years and found a 20% failure rate as well as an increase of higher grade osteoarthritis [27]. Performing a modified Grammont procedure in combination with MPFL reconstruction still leaves the door open for subsequent trochleoplasty. Therefore, an active decision for a less invasive procedure was made and trochleoplasty is considered as a possible secondary procedure in case of unfavorable outcome.

Limitations of the present study include its retrospective character and the small number of included patients. Therefore, a prospective preferably multi-centric study would be necessary to confirm the presented findings. With a median follow-up of 50 months, it is still not possible to predict long-term complications, such as osteoarthritis. Although no osteoarthritic changes were observed in our patients a longer follow-up period is needed to exclude unfavorable sequelae. Nevertheless, the presented results are promising and a modified Grammont technique in combination with MPFL reconstruction might be a valuable alternative to more extensive procedures in adolescent patients with mild trochlear dysplasia and patellar traction malalignment.

## Conclusion

The combination of a modified Grammont technique and MPFL reconstruction yielded favorable results in a small group of adolescents with trochlear dysplasia, elevated TTTG, and recurrent patellar dislocations. No re-dislocations were reported and knee scores improved significantly. The presented approach could help to avoid major procedures, such as osteotomies in a selected group of patients.
